# Down-Regulation of *OsEMF2b* Caused Semi-sterility Due to Anther and Pollen Development Defects in Rice

**DOI:** 10.3389/fpls.2017.01998

**Published:** 2017-11-22

**Authors:** Luchang Deng, Siwei Zhang, Geling Wang, Shijun Fan, Meng Li, Weilan Chen, Bin Tu, Jun Tan, Yuping Wang, Bingtian Ma, Shigui Li, Peng Qin

**Affiliations:** ^1^Rice Research Institute of Sichuan Agricultural University, Chengdu, China; ^2^Crop Research Institute, Sichuan Academy of Agricultural Sciences, Chengdu, China; ^3^State Key Laboratory of Hybrid Rice, Sichuan Agricultural University, Chengdu, China

**Keywords:** rice, *OsEMF2b*, semi-sterility, anther development, male reproductive development

## Abstract

Anther and pollen development are crucial processes of plant male reproduction. Although a number of genes involved in these processes have been identified, the regulatory networks of pollen and anther development are still unclear. *EMBRYONIC FLOWER 2b* (*OsEMF2b*) is important for rice development. Its biological function in floral organ, flowering time and meristem determinacy have been well-studied, but its role, if only, on male reproduction is still unknown, because null mutants of *OsEMF2b* barely have anthers. In this study, we identified a weak allele of *OsEMF2b*, *osemf2b-4*. The T-DNA insertion was located in the promoter region of *OsEMF2b*, and *OsEMF2b* expression was significantly decreased in *osemf2b-4*. The *osemf2b-4* mutant exhibited much more normal anthers than null mutants of *OsEMF2b*, and also showed defective floret development similar to null mutants. Cytological analysis showed various defects of anther wall and pollen development in *osemf2b-4*, such as slightly or extremely enlarged tapetum, irregular or normal morphology microspores, and partial or complete sterility. *OsEMF2b* was highly expressed in tapetum and microspores, and the protein was localized in the nucleus. The expression of 15 genes essential for anther and pollen development was investigated in both WT and *osemf2b-4*. Fourteen genes including *GAMYB* was up-regulated, and only *PTC1* was down-regulated in *osemf2b-4*. This suggests that up-regulated *GAMYB* and down-regulated *PTC1* might contribute to the defective anther and pollen development in *osemf2b-4*. Overall, our work suggests that *OsEMF2b* plays an essential role during post-meiotic anther and pollen development.

## Introduction

Male reproductive development in plants is a complex biological process involving coordinated differentiation of anthers and the generation of gametophytes, then anther dehiscence and subsequently pollination. The anther usually consists of gametophytes at the center, surrounded by the tapetum and the anther wall with the middle cell layer, endothecium, and outer epidermis (Goldberg et al., 1993). During later anther development, major processes include programmed cell death (PCD) of the tapetum, and of the anther wall layers to release mature pollen. These highly coordinated processes require precise fine-tuning of gene regulatory networks such as transcriptional and epigenetic regulation. For example, histone H2B monoubiquitination mediated by HISTONE MONOUBIQUITINATION1 (OsHUB1) and OsHUB2 is involved in anther development by directly regulating tapetum degradation-related genes such as the transcription factor *UNDEVELOPMENT TAPETUM1* (*UDT1*) (Cao et al., 2015). The chromatin protein MALE MEIOCYTE DEATH1 (MMD1) binds to H3K4me2 via the plant homeo domain (PHD) finger domain and this interaction is critical for function during *Arabidopsis* male meiosis (Andreuzza et al., 2015). SET DOMAIN GROUP 4 (SDG4) functions in the maintenance of methylated histone H3K4 and K36 levels in the mature pollen grain (Cartagena et al., 2008). SDG2 mediates H3K4me3 deposition at *SPOROCYTELESS/NOZZLE* (*SPL/NZZ*) and *MALE STERILITY1* (*MS1*) for transcriptional activation, which are essential for differentiation of anther walls and pollen development in *Arabidopsis* (Berr et al., 2010). However, the molecular mechanisms regulating gene expression with histone methylations in gametophyte development remain elusive.

Polycomb group complexes (PcG) are conserved in molecular function and composition between *Drosophila*, mammals and plants, and regulate development through epigenetic control of gene expression via chromatin remodeling (Luo et al., 2009). One of the core polycomb repressive complexes, POLYCOMB REPRESSIVE COMPLEX2 (PRC2) is mainly responsible for tri-methylation of lysine 27 of histone H3, a hallmark of silenced genes in the euchromatin in *Arabidopsis* (Zhang et al., 2007). The EMF2-PRC2 complex composed of EMBRYONIC FLOWER2 (EMF2), FERTILIZATIONINDEPENDENT ENDOSPERM (FIE), MULTICOPY SUPPRESSOR OF IRA1 (MSI1), and CURLY LEAF/SWINGER (CLF/SWN) has been identified in *Arabidopsis* (Kohler and Villar, 2008). Loss of *EMF2* causes direct flowering at the heading stage, passing vegetative shoot growth, but generates abnormal flower organs, and sterile florets (Moon et al., 2003). The EMF2 complex is not only involved in the transition from the vegetative to reproductive phase and flower organ development (Chanvivattana et al., 2004), but is also involved in seed development and stress response, via repression of the target genes such as *ABSCISIC ACID INSENSITIVE3* (*ABI3*) (Kim et al., 2010).

Rice *OsEMF2a* and *OsEMF2b* are two paralogs of *Arabidopsis EMF2*, which are expressed in all tissues (Luo et al., 2009). The T-DNA insertions of *OsEMF2b* exhibited abnormal flowering time and defective flower organs (Luo et al., 2009; Conrad et al., 2014; Xie et al., 2014), although no flowering phenotype was observed in rice when the expression of *OsEMF2a* was enhanced (Li K. et al., 2006). OsEMF2b physically interacted with rice VERNALIZATION INSENSITIVE3-LIKE2 (OsVIL2) and controlled late flowering by increasing expression of *LEAFY COTYEDON 2 AND FUSCA 3-LIKE1* (*OsLFL1*) and decreasing expression of *EARLY HEADING DATE1* (*Ehd1*) (Yang et al., 2013). OsEMF2b also regulated meristem determinacy through modulating the expression of E-function homeotic genes such as *OsMADS34* and the B-function gene *OsMADS4*, via H3K27me3 repression (Conrad et al., 2014; Xie et al., 2014). The null mutants of *OsEMF2b* exhibited very few anthers, therefore, it was difficult to investigate its molecular function during anther and pollen development. Here, we identify a weak function allele of *OsEMF2b*, *osemf2b-4*. The *osemf2b-4* mutant exhibits much more normal anthers than the null mutants of *OsEMF2b*. Cytological analysis shows defective anther wall and pollen development. *OsEMF2b* is highly expressed in anthers, especially in the microspores, tapetum and connectives of anther. OsEMF2b is localized in nucleus, and required for fine-tuning the expression of 15 genes which are essential for anther and pollen development. Down-regulated *PTC1* and up-regulated *GAMYB* possibly contribute to defective anther and pollen development in *osemf2b-4*. Our results suggest that *OsEMF2b* plays an essential role in the regulation of anther and pollen development gene through modulating the expression of genes involved in that process.

## Materials and Methods

### Plant Growth Conditions and Genotyping

Lines PFG_K-03014.R of rice (*Oryza sativa japonica* cv. *KitaaKe*), which carries a T-DNA insertion in LOC_Os09g13630 (*OsEMF2b*), was obtained from RiceGE^1^. All plants were grown in rice paddies at Wenjiang, Sichuan and Lingshui, Hainan, where represent natural long-day and short-day conditions, respectively. Genotypes were determined by PCR using a pair of gene-specific primers listed in **Supplementary Table S2**.

### Characterization of Anther and Pollen Phenotypes

For pollen viability, pollen was stained using I_2_-KI solution and observed under a Zeiss Imager A2 microscope. The panicle photograph was taken using the Canon EOS 60D digital camera and the spikelets were observed using a Zeiss Discovery V20 microscope. Anther development was classified into three stages as in Feng et al. (2000) and Zhang and Wilson (2009) and semi-thin sections were prepared as described (Qin et al., 2013). Briefly, spikelets at various developmental stages were fixed in 3% (v/v) glutaraldehyde in 0.2 M sodium phosphate buffer (pH 7.0), then dehydrated in a graded series of ethanol solutions, embedded in Technovit 7100 resin (Hereaus Kulzer, Germany), polymerized at 45°C, and sectioned at 2 μm thickness using a microtome (Leica RM2235, Germany). The sections were stained with 0.25% (w/v) toluidine blue (Sigma) and photographed using a Nikon E600 microscope.

### RNA *in Situ* Hybridization

Wild type spikelets of different developmental stages were fixed in 5% acetic acid, 50% ethanol, and 3.7% formaldehyde in DEPC water for 16 h at 4°C, then dehydrated through an ethanol series, embedded in Paraplast Plus (Sigma), and sectioned at 8 μm using a microtome (Leica RM2235, Germany). A gene-specific region of *OsEMF2b* was amplified for probe preparation using *OsEMF2b* specific primers, and cloned into a pBluescript vector (TransGen Biotech, China), then digested with *EcoR*I or *BamH*I and transcribed with T7 RNA polymerase using the DIG RNA labeling kit (Roche), for both antisense and sense probes. RNA hybridization and probe detection were performed as described (Zhu et al., 2011).

### RNA Isolation and Real-Time Quantitative Reverse Transcription PCR (RT-qPCR)

Total RNAs were extracted using TRIzol reagent (Invitrogen) from rice leaves and two stages of inflorescences, according to distance from the auricle of 0 or 1 cm, which are the standards for meiosis and early young microspore stage, respectively (Kerim et al., 2003). 1 μg total RNAs were used to synthesized first-strand cDNA using Primescript RT reagent Kit with gDNA Eraser following the manufacturer’s instructions [TaKaRa Biotechnology (Dalian), Co., Ltd, Japan]. Specific primers were designed according for individual genes (**Supplementary Table S2**). First-strand cDNA (2 μl) was amplified with 2 μl of specific primers in a total reaction volume of 20 μl, and each reaction was performed in triplicate using KAPA SYBR FAST qPCR Master Mix (KAPA BIOSYSTEMS) in a Thermal Cycler CFX96 (Bio-Rad, United States). The ubiquitin gene was used as an internal control for data normalization and changes in gene expression were calculated using the 2^ΔΔC_T_^ algorithm (Liu et al., 2012). The means from three biological replicates were used for analysis.

### Subcellular Localization Analysis

mCherry cDNA was amplified from pBIN20-ER-rk (Nelson et al., 2007) with the primer pairs mCherry F and mCherry R, and cloned into pC2300-35S-eGFP vector (Ma et al., 2017) to generate pC2300-mCherry with *Kpn*I and *BamH*I restriction enzyme sites. The coding sequence of *OsEMF2b* was amplified using the primer pairs OsEMF2b-mCherry F and OsEMF2b-mCherry R listed in **Supplementary Table S2**. The amplified fragment and pC2300-mCherry backbone were digested with *BamH*I and *Xba*I, then ligated together to create pC2300-mCherry-OsEMF2b. The pC2300-mCherry and recombinant vector pC2300-mCherry-OsEMF2b were then transformed into rice protoplasts as described (Zhang et al., 2011). mCherry fluorescence was visualized using a ZEISS LSM 710 confocal microscope.

### Complementation of the *osemf2b-4* Mutant

The entire coding region of *OsEMF2b* was amplified with KOD DNA polymerase (TOYOBO, Japan) using the primer pairs OsEMF2b-comp F and OsEMF2b-comp R. The amplified fragment was digested with *Spe*I and *Pst*I and then ligated to pCactN carrying the rice ACTIN promoter (Zhu et al., 2016) to generate pCactN-EMF2b, which was transformed into *osemf2b-4* using *Agrobacterium tumefaciens* (Hiei et al., 1994). Positive transgenic plants were selected with G418 and identified using the primer pair for *Neomycin phosphotransferase II* gene (*NPT II*).

### Statistical Analysis

Statistical significance of differences in the mean values was determined using the Student’s *t*-test function in Microsoft Excel software. Levels of significance were represented by asterisks as follows: ^∗^,^∗∗^ indicates significance at *P* ≤ 0.05 or 0.01, respectively.

## Results

### Identification and Morphological Characterization of *osemf2b-4* Plants

**FIGURE 1:**
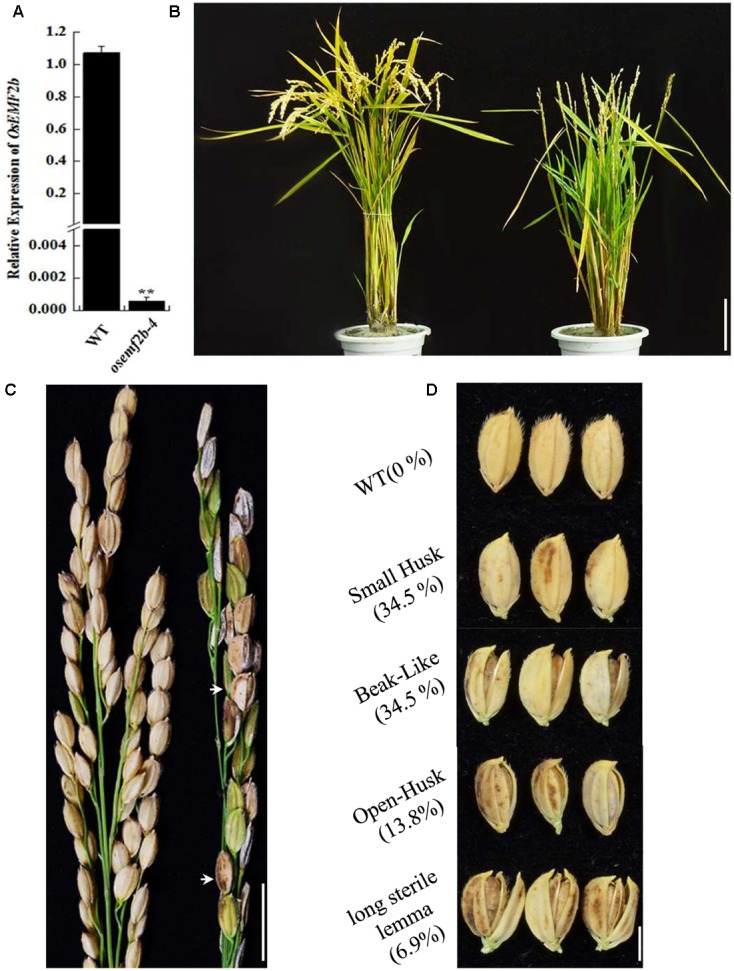
Phenotypic and RT-qPCR analyses of *osemf2b-4.* **(A)** Expression levels of *OsEMF2b* in the WT or *osemf2b-4* leaves were detected by RT-qPCR. Error bars indicate SD. ^∗∗^ indicates significant difference by Student’s *t*-test (*P* ≤ 0.01). **(B)** Plant phenotypes of wild type (left) and *osemf2b-4* (right) at maturity. Bars = 5 cm. **(C)** Panicles of wild type (left) and *osemf2b-4* (right). Abnormal seeds are marked with white arrow in the *osemf2b-4* panicle. Data are given as mean ± SEM (*n* = 3). Bars = 2 cm. **(D)** Seed phenotypes of wild type and *osemf2b-4*. The numbers to the left were calculated as the proportion of slightly defective types of abnormal seeds to spikelets for each plant at maturity. The 10.3% remaining were sterile spikelets. Bars = 5 mm.

Based on RiceGE database and sequencing results, the T-DNA insertion of *osemf2b-4* was inserted -200 bp upstream of the *OsEMF2b* start codon (**Supplementary Figure S1**). RT-qPCR analysis revealed that the expression of *OsEMF2b* was significantly decreased in leaves of *osemf2b-4* (**Figure 1A**), indicating that it was a weaker allele than the three null mutant alleles (Kim et al., 2010). The *osemf2b-4* homozygous mutant was slightly shorter than wild type (**Figure 1B**). The most obvious phenotypes of *osemf2b-4* were reduced grain number and seed set (**Figure 1C**). The grain number per panicle of wild type was 59 ± 3.6, whereas *osemf2b-4* was 22.3 ± 2.1. Additionally, a variety of abnormal seeds were observed, for example, some seeds protruded from the hull, and some had a long sterile lemma (**Figure 1D**).

To test whether the pleiotropic phenotype of *osemf2b-4* was caused by the T-DNA insertion in its promoter region, we firstly carried out co-segregation analysis using *T_1_* and *T_2_* segregation populations (**Supplementary Table S1**). The coding sequence of *OsEMF2b* driven by rice ACTIN promoter was introduced into homogenous *osemf2b-4*, and 4 *T_0_* transgenic plants were obtained (**Supplementary Figure S2**), the 3 with highly enhanced expression of *OsEMF2b* exhibited normal florets, anther and pollen, whereas the one with relatively low expression still exhibited defective floret, anther and pollen development (**Supplementary Figures S2A–C**, **S3A–J**). Together, these results suggested that the T-DNA insertion in *OsEFM2b* promoter region was responsible for the *osemf2b*-4 phenotypes.

### Fertility Analysis of the *osemf2b-4* Mutant

**FIGURE 2:**
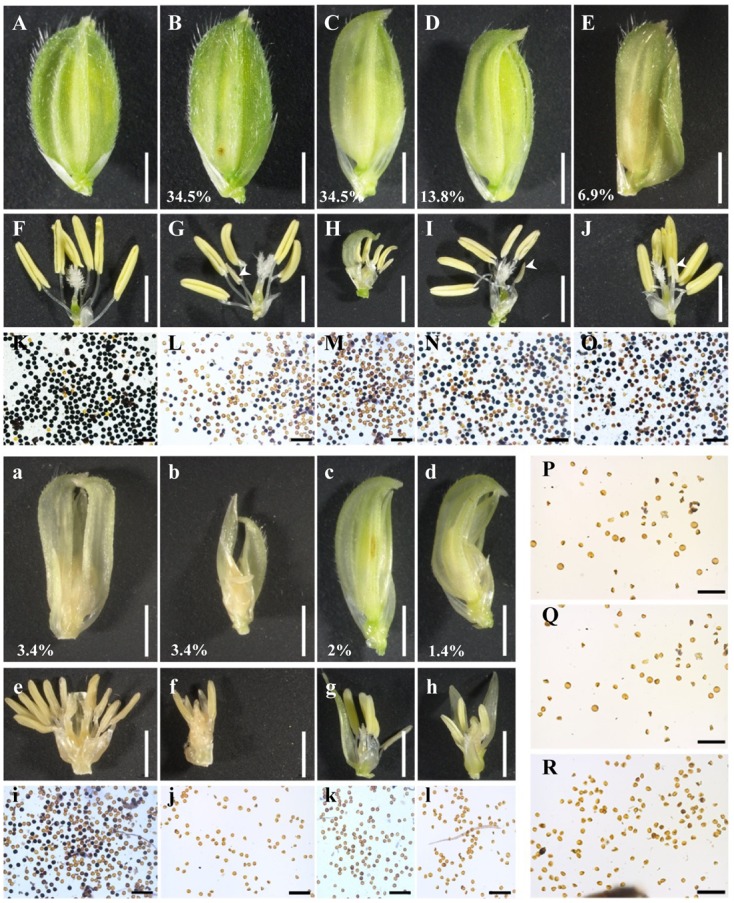
Floret and pollen fertility comparisons of the wild type and *osemf2b-4.* **(A)** A wild type spikelet; **(B–E)** four types of slight floret defects with stamens including small husk **(B)**, beak-like **(C)**, open husk **(D)**, and long sterile lemma **(E)** spikelets in *osemf2b-4* plants, bars = 2 mm; **(F–J)** spikelets after removing the lemma and palea, bars = 2 mm; **(K–R)** I_2_-KI pollen staining, **(L–O)** pollen from yellow anthers, **(P–R)** pollen from three white anthers (white arrowheads), respectively. Bars = 200 μm. **(a–d)** Four types of severe floret defects with stamens such as double lemma **(a)**, coiled lemma and palea **(b)**, missing lemma or palea **(c)**, and leaf-like **(d)** spikelets in *osemf2b-4* mutant, Bars = 2 mm; **(e–h)** spikelets after removing the lemma and palea, bars = 2 mm; **(i–l)** Pollen fertility using I_2_-KI pollen staining. Numbers indicate the ratio of different types of floral organ defects with stamens per plant. Bars = 200 μm.

Previous studies on biological and molecular function of *OsEMF2b* were focused on flowering time, inflorescence and floret development using its null mutant (Luo et al., 2009; Conrad et al., 2014; Xie et al., 2014). Here, we used the weak allele *osemf2b-4* to study its function during post-meiotic anther and pollen development. The spikelets of *osemf2b-4* exhibited various morphologies of palea and/or lemma (**Figure 2A**). We divided these florets with stamen into two different types, slight defects (**Figures 2B–E**) and severe defects (**Figures 2a–d**). In *osemf2b-4*, there was a high percentage of slightly defective types including small husk, beak-like, open husk and long sterile lemma florets types, 34.5, 34.5, 13.8, and 6.9% (*n* = 142), respectively. The most severe cases, such as double lemma (3.4%, *n* = 142), coiled lemma and palea (3.4%, *n* = 142), missing lemma or palea (2%, *n* = 142), and leaf-like floret (1.4%, *n* = 142).

The palea and lemma were removed for clearly visualizing anthers (**Figures 2F–J,e–h**). In *osemf2b-4*, normal anthers were observed in most florets, but the number of anthers in each floret were variable. In the three null mutants anthers were rarely produced but did not contain any pollen grains (Luo et al., 2009; Kim et al., 2010). I_2_-KI staining showed that the pollen fertility of *osemf2b-4* anthers was variable, from semi-sterility to complete sterility (**Figures 2L–O,i–l**), when compared with the fertility of WT anthers (**Figure 2K**). Abortive pollen grains were observed in the transparent and white stamens (**Figures 2P–R**). These results suggested that the partial spikelet sterility of the *osemf2b-4* mutant was likely caused by reduced pollen viability, and that *osemf2b-4* was an excellent allele for studying the function of *OsEMF2b* during post-meiotic anther and pollen development.

### Cytological Characterization of Post-meiotic Anther and Pollen Development in *osemf2b-4*

**FIGURE 3:**
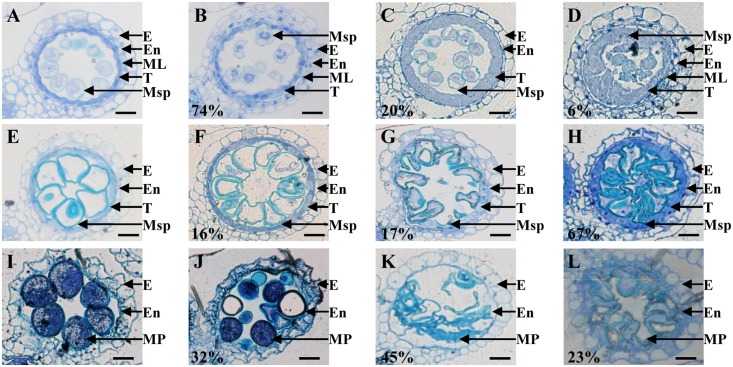
Histological comparison of anther development in the wild type and *osemf2b-4*. Three stages of anther development in the wild type and *osemf2b-4* are compared. The images are of cross sections through a single locule. Wild type sections are shown in **(A,E,I)**, and others show *osemf2b-4* sections. **(A–D)** Young microspore stage; **(E–H)** vacuolated pollen stage; **(I–L)** mature pollen stage. Numbers indicate the percentage of different anther defects at one stage. E, epidermis; En, endothecium; ML, middle layer; T, tapetum; Ms, microsporocyte; Msp, microspore; MP, mature pollen. Bars = 15 μm.

To more precisely determine the cytological reason for the post-meiosis anther and pollen defects in *osemf2b-4*, we performed semi-thin sections for at three stages of anther development (Feng et al., 2000; Zhang and Wilson, 2009). According to the anther defects in different locules, *osemf2b-4* anthers were grouped into three different categories from young microspore stage to mature pollen stage, respectively (**Figures 3A–L**). At the young microspore stage, the tapetal cells was deeply stained, and exine was deposited on the round microspores in wild type anthers (**Figure 3A**). However, three different phenotypes were observed in *osemf2b-4* anthers: type I (74%, *n* = 45) with likely normal tapetum and irregular shape of microspores (**Figure 3B**); type II (20%, *n* = 45) with lightly stained and weakly expanded tapetum and absent middle layer (**Figure 3C**); type III (6%, *n* = 45) with extremely enlarged and disordered tapetum and no exine on microspores (**Figure 3D**). At the vacuolated stage in wild type anthers, the tapetal cells had become more degenerated and the microspores appeared vacuolated (**Figure 3E**). The mutant anthers exhibited three different types, including type I (16%, *n* = 146) with likely normal locules (**Figure 3F**), type II (17%, *n* = 146) with shrunken microspores (**Figure 3G**), and type III (67%, *n* = 146) with delayed degradation of tapetum and folded microspores (**Figure 3H**).

By the mature pollen stage, the tapetum and middle layer had completely degenerated and the endothecial cell layer eventually was disrupted to release pollen grains in wild type (**Figure 3I**). In *osemf2b-4*, 32% of anther locules (*n* = 38) displayed linear shaped surface, similar to wild type anthers. Some pollen grains were stained weakly with toluidine blue, while some grains were irregularly shaped and collapsed in type I (**Figure 3J**). Another two types of anther defects, type II (45%, *n* = 38) and type III (23%, *n* = 38) exhibited glossy and smooth anther surface and shrunken pollen, as well as delayed degradation of the endothecial cell layer (**Figures 3K,L**). Together, these observations suggested that *osemf2b-4* had developmental defects in post-meiotic anther wall layers and microspores, and that *OsEMF2b* plays an essential role in post-meiotic anther and pollen development.

### Expression Pattern of *OsEMF2b* in Anther and Subcellular Localization of OsEMF2b

**FIGURE 4:**
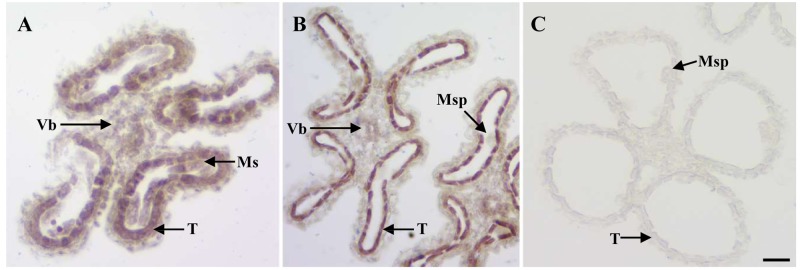
RNA *in situ* analysis of *OsEMF2b.* **(A)** A wild type anther at the meiosis stage showing *OsEMF2b* expression in microsporocytes, tapetum and connectives of anther. **(B)** A wild type anther at the young microspore stage showing stronger *OsEMF2b* expression in tapetal cells, and weaker expression in microspores and connectives of anther. **(C)** A wild type anther at young microspore stage with the sense probe. Ms, microsporocyte; T, tapetum; Msp, microspore; Vb, vascular bundle of anther connective. Bars = 15 μm.

Given the anther defects observed in *osemf2b-4*, we examined the expression pattern of *OsEMF2b* at two developmental stages using *in situ* hybridization (**Figures 4A–C**). *OsEMF2b* transcripts were detected in microsporocyte, tapetum and vascular bundle of anther connective at the meiosis stage (**Figure 4A**). *OsEMF2b* had high expression in tapetum and weak expression in microspores and vascular bundle at the young microspore stage (**Figure 4B**). Only background level of signal was detected with the sense probe (**Figure 4C**).

**FIGURE 5:**
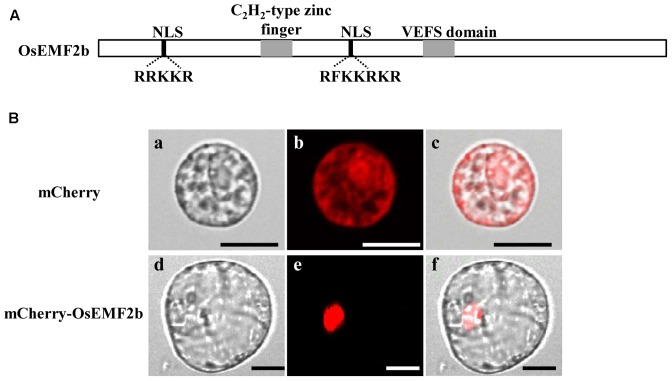
Structure and nuclear localization of OsEMF2b. Schematic representation of OsEMF2b. The black boxes indicate two putative nuclear localization signals (NLS) with a basic segment (Lys-Lys-Arg-Lys-Arg), and the gray boxes indicate a C_2_H_2_-type zinc finger motif and the VRN2-EMF2-FIS2-SUZ12 (VEFS) domain, respectively. **(A)** Nuclear localization of OsEMF2b in rice protoplasts. **(B)** **(a–c)** Images of mCherry. **(d–f)** Images of mCherry-OsEMF2b fusion. **(a,d)** Bright-field images. **(b,e)** Red fluorescence images. **(c,f)** Merged images of **(a,b,d,e)**, respectively. Bars = 2 μm in **(a–c)**, 4 μm in **(d–f)**.

Even though OsEMF2b was predicted to be localized in the nucleus (Yoshida et al., 2001; Luo et al., 2009), due to two putative nuclear localization signals, a single C_2_H_2_-type zinc finger motif and a VRN2-EMF2-FIS2-SUZ12 (VEFS) domain (**Figure 5A**), no experimental data showed its protein localization. In order to test the subcellular localization of OsEMF2b, a 35S::mCherry-OsEMF2b fusion construct was introduced into rice leaf protoplasts. As expected, the mCherry-OsEMF2b fusion protein was observed in the nucleus (**Figures 5Bd–f**), whereas the empty vector with 35S::mCherry was observed in cytoplasm (**Figures 5Ba–c**).

### Expression of Genes Involved in Anther and Pollen Development Was Affected in *osemf2b-4*

**FIGURE 6:**
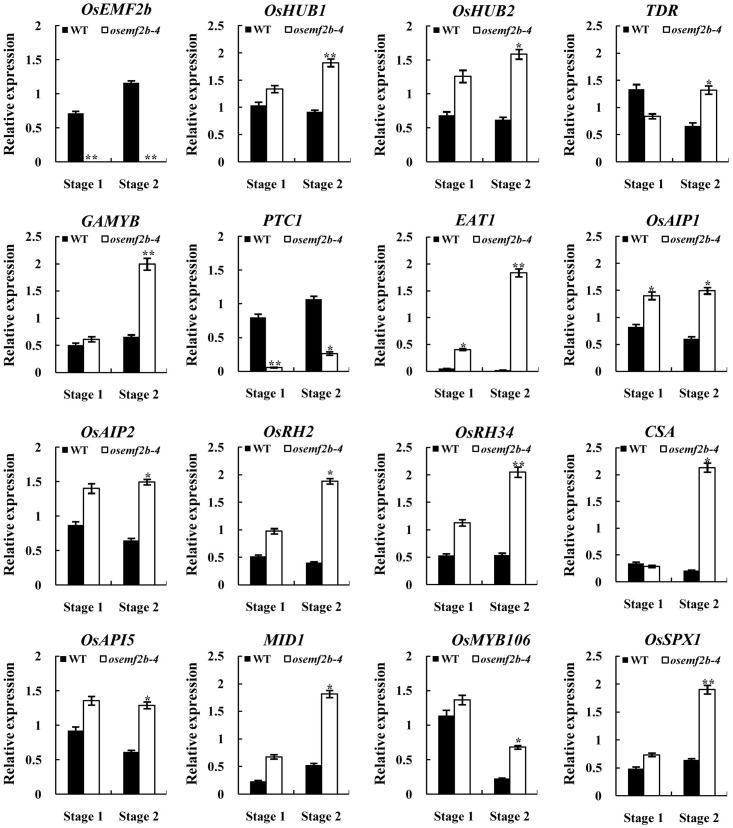
RT-qPCR expression analysis of *OsEMF2b* and genes involved in rice anther and pollen development in the inflorescence of the wild type and *osemf2b-4* plants. Stage 1 and stage 2 represent meiosis stage and young microspore stage, respectively. The ubiquitin gene was used as an internal control. Each data point is the average of three biological repeats. Error bars indicate SD. ^∗^,^∗∗^ indicate significant difference by Student’s *t*-test (*P* ≤ 0.05 and 0.01).

Defective anther development was observed in *osemf2b-4*, probably by affecting the gene expressions involved in post-meiotic anther and pollen development. We used the auricle distance between the penultimate leaf and flag leaf for assessing anther development stage; 0 and 1 cm respectively represented meiosis and young microspore stage (Kerim et al., 2003). Then we analyzed the expression of several nuclear genes involved in anther and pollen development at these two stages. Most anther and pollen-related genes had tissue-specific expression patterns. *TAPETUM DEGENERATION RETARDATION* (*TDR*) (Li N. et al., 2006) and *ETERNAL TAPETUM1* (*EAT1*) (Niu et al., 2013) were preferentially expressed in the tapetum, as *HISTONE MONOUBIQUITINATION1/2* (*OsHUB1/2*) (Cao et al., 2015), *GAMYB* (Aya et al., 2009), *APPOTOSIS INHIBITOR5* (*OsAPI5*), *OsAIP1/2* (Li X. et al., 2011), *CARBON STARVED ANTHER* (*CSA*) (Zhu et al., 2015), *MYB IMPORTANT FOR DROUGHT RESPONSE1* (*MID1*) (Guo et al., 2016) and *PERSISTENT TAPETAL CELL1* (*PTC1*) (Li H. et al., 2011) were expressed in the tapetum and microspores. *OsRH2/34* (Huang et al., 2016), *OsMYB106* and *OsSPX1* (Zhang et al., 2016) were also essential for anther and/or pollen development. *PTC1* expression was significantly decreased in *osemf2b-4*, and the other 14 genes were significantly up-regulated at stage 2 (**Figure 6**), suggesting that *OsEMF2b* was required for fine-tuning the expression of these genes involved in the anther and pollen development.

## Discussion

In this study, we identified a weak *OsEMF2b* mutant, *osemf2b-4*, which exhibited defective floret development and seed set. Cytological analysis showed various defects during anther and pollen development in *osemf2b-4*, such as delayed tapetum degradation. RNA *in situ* showed that *OsEMF2b* was expressed in the tapetum and microspores. These results suggested that *OsEMF2b* plays an important role in anther and pollen development.

*EMF2* played an important role in repression of genes by promoting H3K27me3 at target genes in *Arabidopsis* (Conrad et al., 2014). We therefore investigated the expression of 15 genes whose encoded proteins are nuclearly localized and are essential for anther and pollen development. Among these genes, the expression of *PTC1* was significantly decreased in *osemf2b-4*. *PTC1* plays an essential role in rice anther and pollen development, as a knockout (Li H. et al., 2011) or knock-down (results not published) of *PTC1* resulted in developmental defects in anthers and pollen. Therefore, the down-regulated expression of *PTC1* in *osemf2b-4* possibly played an important contribution to the defective anther and pollen development observed in *osemf2b-4*. On the other hand, OsEMF2b physically interacts with VERNALIZATION INSENSITIVE3-LIKE2 (OsVIL2), a PHD-finger domain protein showing homologies to the *Arabidopsis* VIN3-group proteins, to promote H3K27me3 deposition in rice (Yang et al., 2013). The *Arabidopsis* VIN3-group proteins with PHD-finger domain are known to work together with the PRC2 core complex containing EMF2 for constituting the so-called PHD-PRC2 complexes (Ietswaart et al., 2012). *PTC1* encodes a PHD-finger protein and its ortholog protein in *Arabidopsis*, MALE STERILITY1 (MS1), contains an aromatic cage required for H3K4me2/3 binding (Lee et al., 2009). Therefore, OsEMF2b and PTC1 might function together through histone methylation modification in anther and microspore development. *PTC1* is expressed in tapetum and microspores at the young microspore stage (Li H. et al., 2011), and the overlapped spatiotemporal expression pattern with *OsEMF2b* (**Figure 4**) are consistent with the idea that OsEMF2b and PTC1 function together.

Considering these 14 genes with up-regulated expression in *osemf2b-4*, all mutants of them exhibited anther and/or pollen developmental defects (Cao et al., 2015; Shi et al., 2015; Huang et al., 2016; Zhang et al., 2016). However, it was unknown whether the over-expression of these 14 genes caused defects in anther and pollen development process in rice. But in barley, over-expressing *HvGAMYB* resulted in partial or complete sterility (Murray et al., 2003). As the molecular pathway of anther development is conserved between rice and barley (Fernández Gómez and Wilson, 2014), it is not surprising that over-expressing *GAMYB* in rice led to anther and pollen developmental defects, and possibly contributes to the phenotype of *osemf2b-4*. As similar with *GAMYB*, both knockout and over-expressed *MS1* exhibited defective anther and pollen in *Arabidopsis* (Yang et al., 2007), indicating that the expression of genes involved in anther and pollen development was tightly controlled. Based on that, these 14 genes with up-regulated expression in *osemf2b-4* should be also tightly controlled in wild type, in order to keep normal expression level to function normally. This tight control in anther and pollen could be done directly or indirectly by OsEMF2b. Overall, our studies suggested that OsEMF2b played an essential role in anther and pollen development, and possibly by fine-tuning the expression of genes expressed in tapetum and microspores, such as *PTC1* and *GAMYB*.

## Author Contributions

LD performed most of the experiments and wrote the manuscript. SZ and ML performed RT-qPCR analyses and complementation test. GW performed the semi-thin sections. SF, WC, and JT finished the subcellular localization analysis. BT provided technical assistance. PQ supervised and complemented the writing. YW, BM, and SL supervised the research.

## Conflict of Interest Statement

The authors declare that the research was conducted in the absence of any commercial or financial relationships that could be construed as a potential conflict of interest.

## Footnotes

1 http://signal.salk.edu/cgi-bin/RiceGE

## Supplementary Material

FIGURE S1 Schematic characterization of four independent mutant alleles of *OsEMF2b*. Three mutants are T-DNA insertions including *osemf2b-1* in intron 19*, osemf2b-2* in intron 10 and *osemf2b-4* in this study. The *osemf2b-3* mutant is generated by targeting induced local lesions in genomes (TILLIING), and is a guanine to thymine substitution.

FIGURE S2 Phenotype **(A,B)** and corresponding relative expression levels **(C)** of *osemf2b-4* and complementation plants at heading stage in *T_0_* progenies. **(A)** Plant phenotypic comparison. Bars = 10 cm. **(B)** Observation of inflorescence. Bars = 2 cm. Comp, complementation plants. The white arrows indicated abnormal spikelets. ^∗∗^ indicates significant difference by Student’s *t*-test (*P* ≤ 0.01).

FIGURE S3 Spikelets after removing the lemma and palea **(A–E)** and I_2_-KI pollen staining **(F–J)** of *osemf2b-4* and complementation plants. The numbers are percentage of stained pollen grains. Bars = 2 mm and 200 μm, respectively.

TABLE S1 Segregation of the *osemf2b-4* lines in *T_1_* and *T_2_* progenies.

TABLE S2 Primers used in this study.
