# Supplementary figures and images for: Down-Regulation of OsEMF2b Caused Semi-sterility Due to Anther and Pollen Development Defects in Rice
Source: Front Plant Sci. 2017 Nov 22;8:1998. doi: 10.3389/fpls.2017.01998 (PMC5715369; doi:10.3389/fpls.2017.01998)

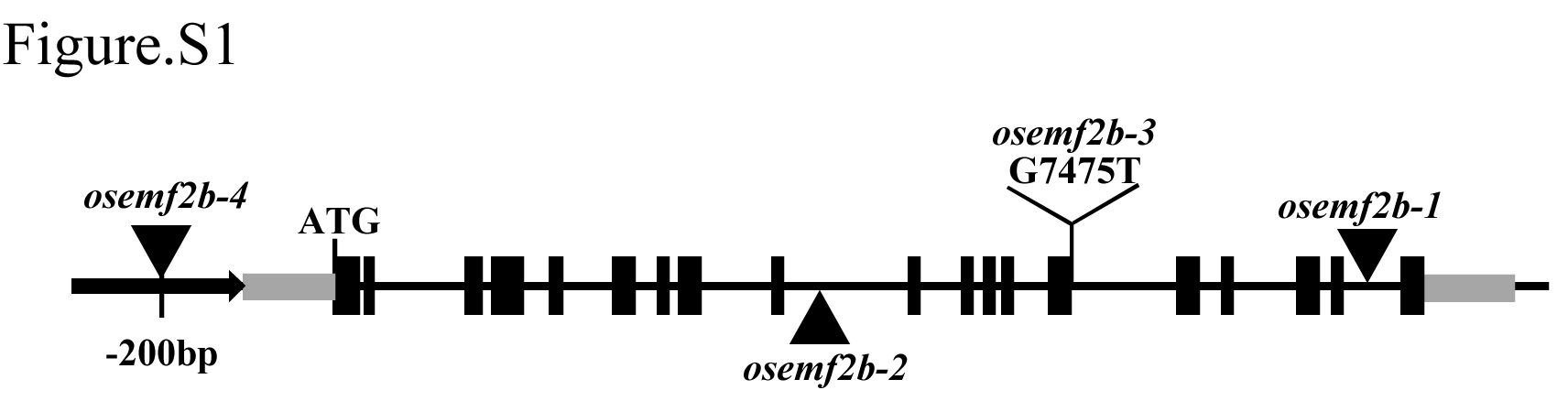

Supplement: FIGURE S1 — Schematic characterization of four independent mutant alleles of OsEMF2b. Three mutants are T-DNA insertions including osemf2b-1 in intron 19, osemf2b-2 in intron 10 and osemf2b-4 in this study. The osemf2b-3 mutant is generated by targeting induced local lesions in genomes (TILLIING), and is a guanine to thymine substitution. [file Image_1.jpeg]

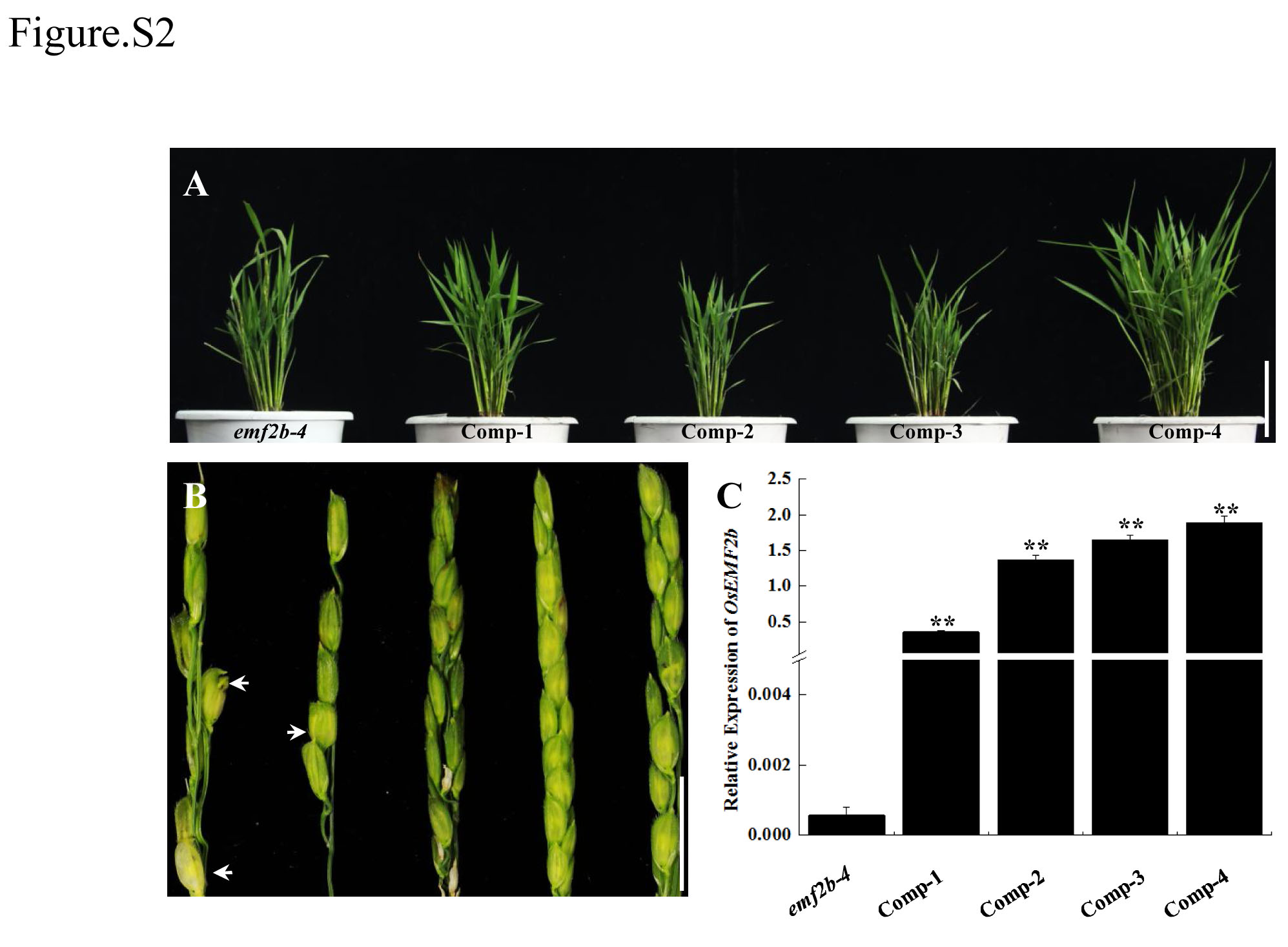

Supplement: FIGURE S2 — Phenotype (A,B) and corresponding relative expression levels (C) of osemf2b-4 and complementation plants at heading stage in T0 progenies. (A) Plant phenotypic comparison. Bars = 10 cm. (B) Observation of inflorescence. Bars = 2 cm. Comp, complementation plants. The white arrows indicated abnormal spikelets. ∗∗ indicates significant difference by Student’s t-test (P ≤ 0.01). [file Image_2.jpeg]

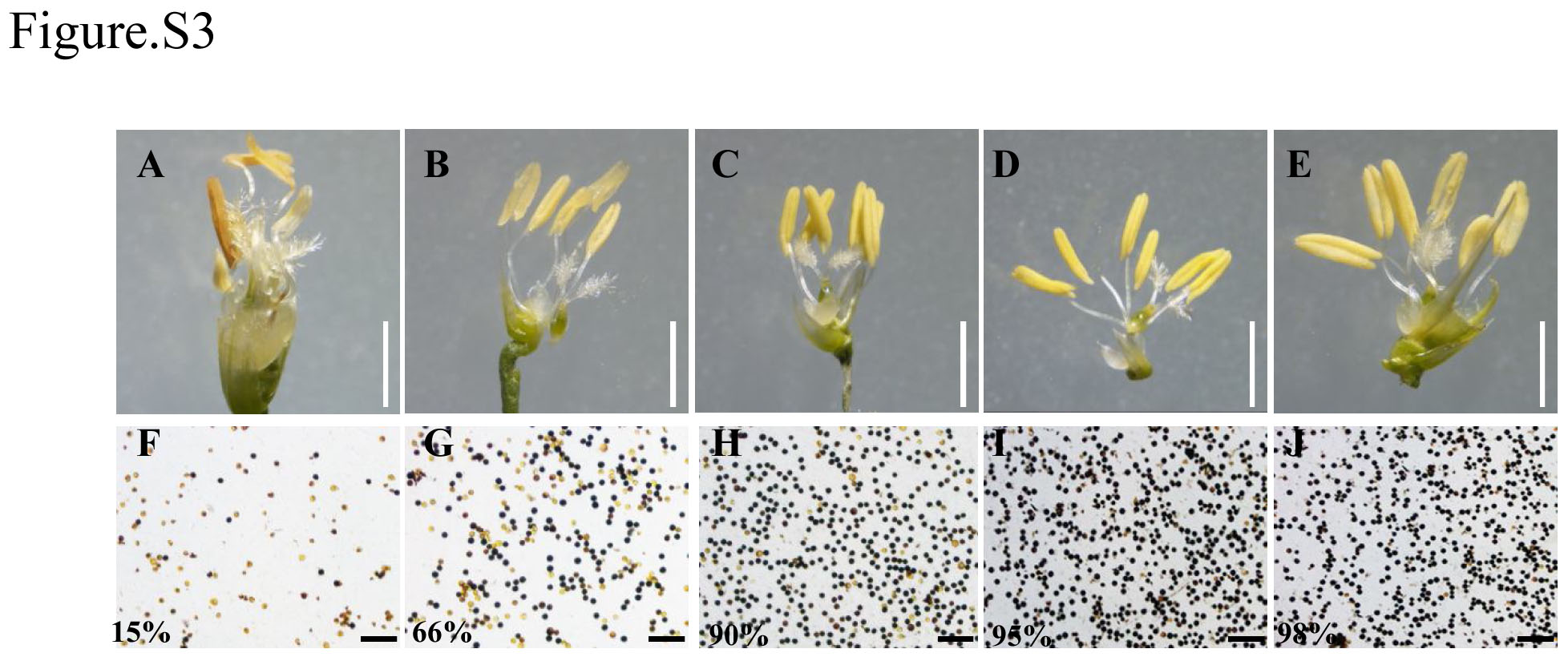

Supplement: FIGURE S3 — Spikelets after removing the lemma and palea (A–E) and I2-KI pollen staining (F–J) of osemf2b-4 and complementation plants. The numbers are percentage of stained pollen grains. Bars = 2 mm and 200 μm, respectively. [file Image_3.jpeg]

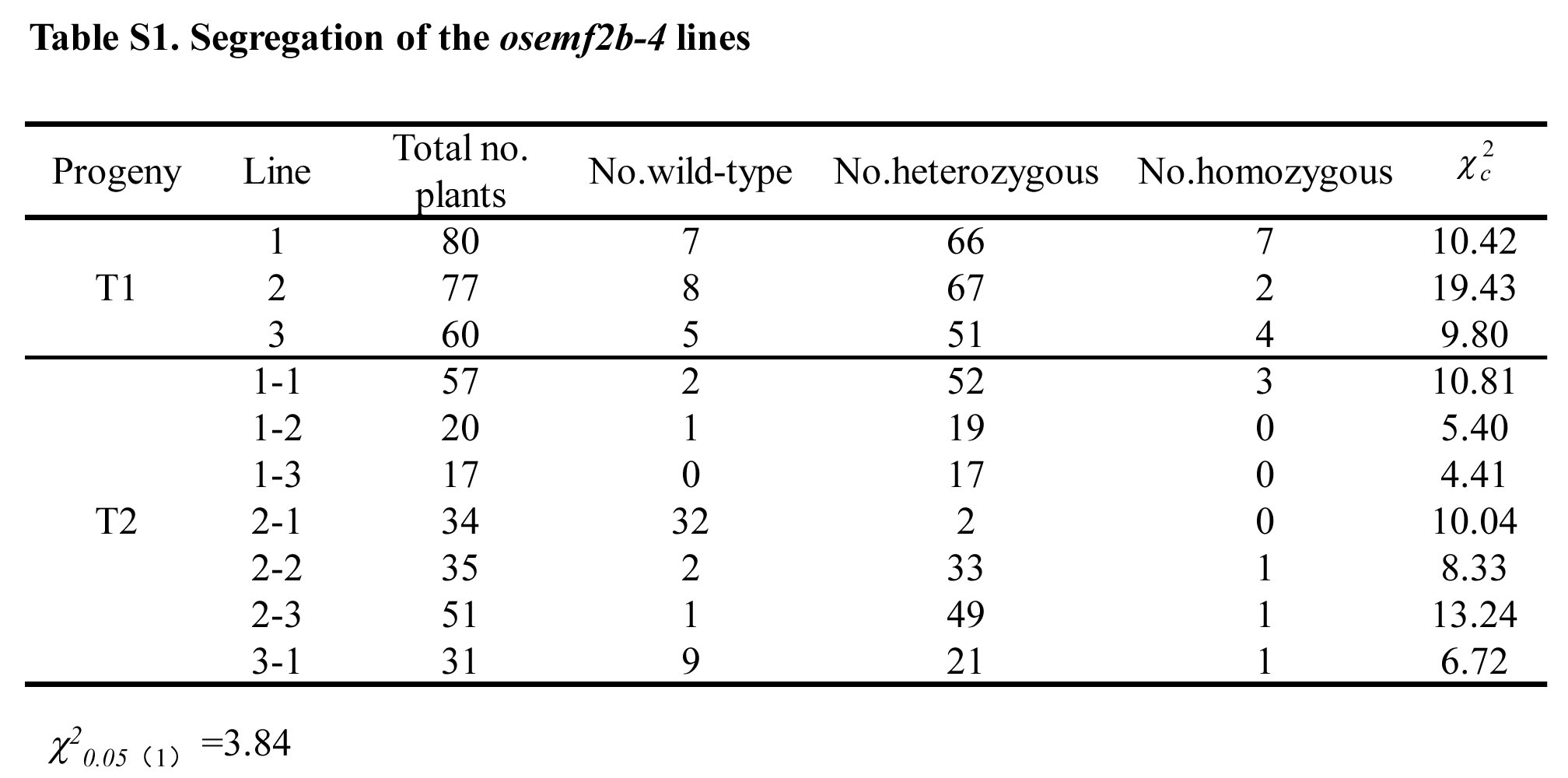

Supplement: TABLE S1 — Segregation of the osemf2b-4 lines in T1 and T2 progenies. [file Table_1.jpeg]
